# 2-Methyl-3-(phenyl­sulfon­yl)naphtho[1,2-*b*]furan

**DOI:** 10.1107/S1600536808000895

**Published:** 2008-01-16

**Authors:** Hong Dae Choi, Pil Ja Seo, Byeng Wha Son, Uk Lee

**Affiliations:** a Department of Chemistry, Dongeui University, San 24 Kaya-dong, Busanjin-gu, Busan 614-714, Republic of Korea; b Department of Chemistry, Pukyong National University, 599-1 Daeyeon 3-dong, Nam-gu, Busan 608-737, Republic of Korea

## Abstract

In the title mol­ecule, C_19_H_14_O_3_S, the phenyl ring forms a dihedral angle of 69.13 (6)° with the plane of the naphthofuran fragment, being slightly tilted towards it. The crystal packing exhibits π–π inter­actions between the benzene rings from neighbouring mol­ecules [centroid–centroid distance = 3.616 (4) Å] and weak C—H⋯O and C—H⋯π inter­actions.

## Related literature

The crystal structure of 2-methyl-3-(methyl­sulfin­yl)naphtho[1,2-*b*]furan has been reported by Choi *et al.* (2006 ▶).
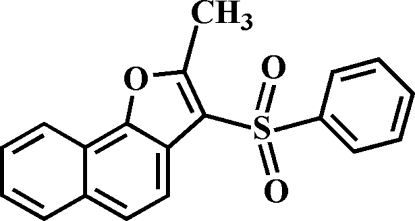


## Experimental

### 

#### Crystal data

C_19_H_14_O_3_S
*M* *_r_* = 322.36
Orthorhombic, 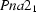

*a* = 8.198 (4) Å
*b* = 18.589 (8) Å
*c* = 10.049 (4) Å
*V* = 1531.4 (11) Å^3^
*Z* = 4
Mo *K*α radiation
μ = 0.22 mm^−1^
*T* = 173 (2) K
0.40 × 0.30 × 0.20 mm

#### Data collection

Bruker SMART CCD diffractometer
Absorption correction: none
8102 measured reflections
2714 independent reflections
2538 reflections with *I* > 2σ(*I*)
*R*_int_ = 0.048

#### Refinement

*R*[*F* ^2^ > 2σ(*F* ^2^)] = 0.031
*wR*(*F* ^2^) = 0.083
*S* = 1.05
2714 reflections
208 parameters
1 restraint
H-atom parameters constrained
Δρ_max_ = 0.26 e Å^−3^
Δρ_min_ = −0.21 e Å^−3^
Absolute structure: Flack (1983 ▶), 1125 Friedel pairs
Flack parameter: 0.04 (7)

### 

Data collection: *SMART* (Bruker, 1997 ▶); cell refinement: *SAINT* (Bruker, 1997 ▶); data reduction: *SAINT*; program(s) used to solve structure: *SHELXS97* (Sheldrick, 2008 ▶); program(s) used to refine structure: *SHELXL97* (Sheldrick, 2008 ▶); molecular graphics: *ORTEP-3* (Farrugia, 1997 ▶) and *DIAMOND* (Brandenburg, 1998 ▶); software used to prepare material for publication: *SHELXL97*.

## Supplementary Material

Crystal structure: contains datablocks global, I. DOI: 10.1107/S1600536808000895/cv2379sup1.cif

Structure factors: contains datablocks I. DOI: 10.1107/S1600536808000895/cv2379Isup2.hkl

Additional supplementary materials:  crystallographic information; 3D view; checkCIF report

## Figures and Tables

**Table 1:** Selected interatomic distances (Å) *Cg*2 and *Cg*3 are the centroids of the C2–C5/C10/C11 benzene ring and the C5–C10 benzene ring, respectively.

*Cg*2⋯*Cg*3^i^	3.616 (4)

Symmetry code: (i) 
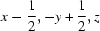
.

**Table 2:** Hydrogen-bond geometry (Å, °) *Cg*1 is the centroid of the O1/C12/C1/C2/C11 furan ring.

*D*—H⋯*A*	*D*—H	H⋯*A*	*D*⋯*A*	*D*—H⋯*A*
C13—H13*A*⋯*Cg*1^i^	0.98	2.64	3.483 (3)	144
C8—H8⋯O3^ii^	0.95	2.51	3.406 (3)	157
C16—H16⋯O3^iii^	0.95	2.51	3.430 (3)	164

Symmetry codes: (i) 
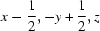
; (ii) 
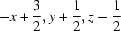
; (iii) 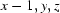.

**Fig. 1.**
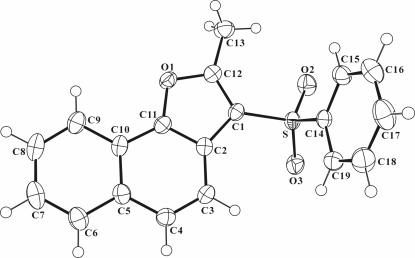
The molecular structure of the title compound, showing displacement ellipsoides drawn at the 50% probability level.

**Fig. 2.**
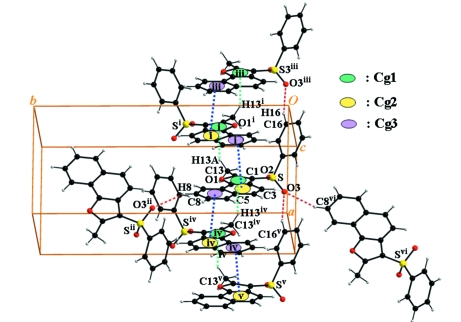
π—π, C—H···π and C—H···O interactions (dotted lines) in the title compound [symmetry codes: (i) x - 1/2, -y + 1/2, z; (ii) -x + 3/2, y + 1/2, z - 1/2; (iii) x - 1, y, z; (iv) x + 1/2, -y + 1/2, z; (v) x + 1, y, z; (vi) -x + 3/2, y - 1/2, z + 1/2.]

## supplementary crystallographic information

### Comment

As part of our ongoing study of 2-methylnaphtho[1,2-*b*]furan derivatives, the crystal structure of 2-methyl-3-(methylsulfinyl)naphtho[1,2-*b*]furan has been recently reported (Choi *et al.*, 2006). Herein we present the molecular and crystal structure of the title compound, (I).

In (I) (Fig. 1), the naphthofuran unit is essentially planar, with a mean deviation of 0.007 Å from the least-squares plane defined by the thirteen constituent atoms. The crystal packing (Fig. 2) is stabilized by aromatic π—π stacking interactions between adjacent benzene rings. The *Cg*2···*Cg*3^i^ distance is 3.616 (4) Å (Table 1; *Cg*2 and *Cg*3 are the centroids of the C2—C5/C10/C11 benzene ring and the C5—C10 benzene ring, respectively, symmetry code as in Fig. 2). The molecular packing is further stabilized by CH_2_—H···π interactions between the methyl group and the furan ring of the naphthofuran unit, with a C13—H13A···*Cg*1^i^ separation of 2.64 Å (Fig. 2 and Table 2; *Cg*1 is the centroid of the O1/C12/C1/C2/C11 furan ring; symmetry code as in Fig. 2). Additionally, the weak hydrogen bonds were observed; one between the benzene H atom of naphthofuran unit and the O atom of sulfonyl group, with a C8—H8···O3^ii^, a second between the benzene H atom of phenylsulfonyl group and adjacent O atom of sulfonyl group, with a C16—H16···O3^iii^ (Fig. 2 and Table 2; symmetry code as in Fig. 2).

### Experimental

3-Chloroperbenzoic acid (77%, 560 mg, 2.5 mmol) was added in small portions to a stirred solution of 2-methyl-3-(phenylsulfanyl)naphtho[1,2-*b*] furan (348 mg, 1.2 mmol) in dichloromethane (40 ml) at 273 K. After being stirred at room temperature for 4 h, the mixture was washed with saturated sodium bicarbonate solution and the organic layer was separated, dried over magnesium sulfate, filtered and concentrated in vacuum. The residue was purified by column chromatography (hexane-ethyl acetate, 2:1 *v*/*v*) to afford the title compound as a pale yellow solid [yield 84%, m.p. 412–413 K; *R*_f_ = 0.64 (hexane-ethyl acetate, 2:1 *v*/*v*)]. Single crystals suitable for X-ray diffraction were prepared by slow evaporation of a dilute solution of the title compound in acetone at room temperature.

### Refinement

All H atoms were positioned geometrically and refined using a riding model, with C—H = 0.95 Å for aromatic H atoms and 0.98 Å for methyl H atoms, respectively, and with *U*_iso_(H) = 1.2Ueq(C) for all H atoms.

### Crystal data

**Table:**

C_19_H_14_O_3_S	*F*_000_ = 672
*M**_r_* = 322.36	*D*_x_ = 1.398 Mg m^−^^3^
Orthorhombic, *P**n**a*2_1_	Mo *K*α radiation λ = 0.71073 Å
Hall symbol: P 2c -2n	Cell parameters from 5173 reflections
*a* = 8.198 (4) Å	θ = 2.2–28.1º
*b* = 18.589 (8) Å	µ = 0.22 mm^−^^1^
*c* = 10.049 (4) Å	*T* = 173 (2) K
*V* = 1531.4 (11) Å^3^	Block, yellow
*Z* = 4	0.40 × 0.30 × 0.20 mm

### Data collection

**Table:**

Bruker SMART CCD diffractometer	2714 independent reflections
Radiation source: fine-focus sealed tube	2538 reflections with *I* > 2σ(*I*)
Monochromator: graphite	*R*_int_ = 0.048
Detector resolution: 10.0 pixels mm^-1^	θ_max_ = 26.0º
*T* = 173(2) K	θ_min_ = 2.2º
φ and ω scans	*h* = −7→10
Absorption correction: none	*k* = −22→22
8102 measured reflections	*l* = −12→11

### Refinement

**Table:**

Refinement on *F*^2^	Hydrogen site location: inferred from neighbouring sites
Least-squares matrix: full	H-atom parameters constrained
*R*[*F*^2^ > 2σ(*F*^2^)] = 0.031	*w* = 1/[σ^2^(*F*_o_^2^) + (0.0507*P*)^2^ + 0.1234*P*] where *P* = (*F*_o_^2^ + 2*F*_c_^2^)/3
*wR*(*F*^2^) = 0.083	(Δ/σ)_max_ < 0.001
*S* = 1.05	Δρ_max_ = 0.26 e Å^−^^3^
2714 reflections	Δρ_min_ = −0.21 e Å^−^^3^
208 parameters	Extinction correction: none
1 restraint	Absolute structure: Flack (1983), 1125 Friedel pairs
Primary atom site location: structure-invariant direct methods	Flack parameter: 0.04 (7)
Secondary atom site location: difference Fourier map	

### Special details

**Table:**

Geometry. All e.s.d.'s (except the e.s.d. in the dihedral angle between two l.s. planes) are estimated using the full covariance matrix. The cell e.s.d.'s are taken into account individually in the estimation of e.s.d.'s in distances, angles and torsion angles; correlations between e.s.d.'s in cell parameters are only used when they are defined by crystal symmetry. An approximate (isotropic) treatment of cell e.s.d.'s is used for estimating e.s.d.'s involving l.s. planes.
Refinement. Refinement of *F*^2^ against ALL reflections. The weighted *R*-factor *wR* and goodness of fit *S* are based on *F*^2^, conventional *R*-factors *R* are based on *F*, with *F* set to zero for negative *F*^2^. The threshold expression of *F*^2^ > σ(*F*^2^) is used only for calculating *R*-factors(gt) *etc*. and is not relevant to the choice of reflections for refinement. *R*-factors based on *F*^2^ are statistically about twice as large as those based on *F*, and *R*- factors based on ALL data will be even larger.

### Fractional atomic coordinates and isotropic or equivalent isotropic displacement parameters (Å^2^)

**Table:**

	*x*	*y*	*z*	*U*_iso_*/*U*_eq_	
S1	0.44268 (6)	0.07870 (2)	0.60161 (6)	0.02617 (13)	
O1	0.51082 (18)	0.27348 (7)	0.46110 (15)	0.0291 (3)	
O2	0.3904 (2)	0.09804 (9)	0.73361 (15)	0.0373 (4)	
O3	0.58124 (17)	0.03221 (8)	0.58544 (17)	0.0369 (4)	
C1	0.4858 (2)	0.15710 (10)	0.5141 (2)	0.0253 (4)	
C2	0.5822 (2)	0.16115 (11)	0.3939 (2)	0.0260 (4)	
C3	0.6620 (3)	0.11149 (11)	0.3089 (2)	0.0293 (4)	
H3	0.6565	0.0612	0.3254	0.035*	
C4	0.7466 (3)	0.13772 (11)	0.2031 (2)	0.0314 (5)	
H4	0.8012	0.1048	0.1460	0.038*	
C5	0.7564 (3)	0.21297 (11)	0.1745 (2)	0.0296 (4)	
C6	0.8432 (3)	0.23949 (14)	0.0629 (2)	0.0379 (5)	
H6	0.8960	0.2067	0.0045	0.045*	
C7	0.8521 (3)	0.31235 (14)	0.0381 (3)	0.0448 (6)	
H7	0.9111	0.3293	−0.0370	0.054*	
C8	0.7749 (3)	0.36149 (12)	0.1225 (3)	0.0421 (6)	
H8	0.7828	0.4115	0.1042	0.051*	
C9	0.6884 (3)	0.33880 (11)	0.2309 (2)	0.0342 (5)	
H9	0.6355	0.3726	0.2872	0.041*	
C10	0.6786 (3)	0.26389 (11)	0.2582 (2)	0.0276 (4)	
C11	0.5932 (2)	0.23396 (10)	0.3665 (2)	0.0270 (4)	
C12	0.4468 (2)	0.22595 (11)	0.5500 (2)	0.0282 (4)	
C13	0.3579 (3)	0.25867 (13)	0.6634 (2)	0.0375 (5)	
H13A	0.2672	0.2878	0.6297	0.045*	
H13B	0.4325	0.2893	0.7143	0.045*	
H13C	0.3154	0.2206	0.7211	0.045*	
C14	0.2753 (2)	0.03853 (10)	0.51984 (19)	0.0249 (4)	
C15	0.1181 (3)	0.05753 (12)	0.5593 (2)	0.0312 (5)	
H15	0.1021	0.0928	0.6265	0.037*	
C16	−0.0141 (3)	0.02474 (14)	0.5000 (2)	0.0404 (5)	
H16	−0.1217	0.0368	0.5272	0.048*	
C17	0.0105 (3)	−0.02571 (14)	0.4009 (3)	0.0469 (6)	
H17	−0.0805	−0.0486	0.3604	0.056*	
C18	0.1664 (3)	−0.04291 (13)	0.3605 (3)	0.0447 (6)	
H18	0.1818	−0.0767	0.2908	0.054*	
C19	0.3008 (3)	−0.01150 (11)	0.4204 (2)	0.0327 (5)	
H19	0.4082	−0.0241	0.3937	0.039*	

### Atomic displacement parameters (Å^2^)

**Table:**

	*U*^11^	*U*^22^	*U*^33^	*U*^12^	*U*^13^	*U*^23^
S1	0.0274 (2)	0.0262 (2)	0.0250 (2)	−0.00075 (19)	−0.0024 (2)	0.0041 (2)
O1	0.0359 (8)	0.0213 (6)	0.0301 (8)	0.0009 (6)	0.0004 (6)	−0.0021 (6)
O2	0.0459 (9)	0.0419 (8)	0.0241 (8)	−0.0059 (8)	−0.0011 (7)	0.0014 (7)
O3	0.0300 (8)	0.0329 (7)	0.0477 (11)	0.0043 (6)	−0.0026 (7)	0.0125 (8)
C1	0.0254 (9)	0.0224 (9)	0.0282 (11)	−0.0009 (8)	−0.0043 (8)	0.0008 (8)
C2	0.0254 (10)	0.0265 (10)	0.0261 (10)	−0.0022 (8)	−0.0030 (8)	0.0021 (8)
C3	0.0337 (11)	0.0226 (9)	0.0317 (11)	−0.0016 (9)	−0.0024 (9)	−0.0003 (8)
C4	0.0356 (12)	0.0298 (10)	0.0290 (11)	0.0004 (9)	0.0012 (9)	−0.0030 (9)
C5	0.0321 (11)	0.0337 (11)	0.0229 (10)	−0.0027 (9)	−0.0040 (8)	0.0025 (9)
C6	0.0411 (13)	0.0454 (14)	0.0271 (12)	−0.0046 (11)	−0.0001 (9)	0.0043 (9)
C7	0.0469 (14)	0.0538 (15)	0.0337 (13)	−0.0117 (12)	−0.0025 (11)	0.0163 (11)
C8	0.0503 (14)	0.0320 (11)	0.0441 (15)	−0.0122 (10)	−0.0105 (11)	0.0122 (11)
C9	0.0381 (12)	0.0278 (10)	0.0366 (13)	−0.0050 (9)	−0.0084 (9)	0.0021 (9)
C10	0.0295 (11)	0.0272 (10)	0.0260 (10)	−0.0050 (8)	−0.0080 (8)	0.0046 (8)
C11	0.0287 (10)	0.0237 (10)	0.0286 (11)	−0.0013 (8)	−0.0042 (8)	−0.0021 (9)
C12	0.0280 (10)	0.0273 (10)	0.0294 (10)	−0.0001 (9)	−0.0032 (8)	−0.0012 (8)
C13	0.0426 (14)	0.0349 (12)	0.0349 (12)	0.0055 (10)	0.0026 (10)	−0.0042 (10)
C14	0.0282 (10)	0.0235 (9)	0.0229 (11)	−0.0014 (8)	0.0004 (8)	0.0052 (8)
C15	0.0327 (11)	0.0338 (10)	0.0271 (11)	0.0014 (10)	0.0036 (8)	0.0036 (8)
C16	0.0297 (11)	0.0534 (14)	0.0380 (14)	−0.0038 (11)	0.0007 (10)	0.0068 (11)
C17	0.0412 (14)	0.0608 (16)	0.0386 (14)	−0.0207 (13)	−0.0045 (11)	0.0009 (12)
C18	0.0576 (15)	0.0430 (13)	0.0335 (13)	−0.0117 (12)	0.0037 (11)	−0.0098 (11)
C19	0.0354 (12)	0.0311 (11)	0.0316 (11)	−0.0021 (9)	0.0058 (9)	−0.0032 (9)

### Geometric parameters (Å, °)

**Table:**

S1—O3	1.437 (2)	C8—C9	1.367 (3)
S1—O2	1.440 (2)	C8—H8	0.9500
S1—C1	1.738 (2)	C9—C10	1.422 (3)
S1—C14	1.765 (2)	C9—H9	0.9500
O1—C12	1.361 (3)	C10—C11	1.409 (3)
O1—C11	1.378 (2)	C12—C13	1.483 (3)
C1—C12	1.368 (3)	C13—H13A	0.9800
C1—C2	1.446 (3)	C13—H13B	0.9800
C2—C11	1.384 (3)	C13—H13C	0.9800
C2—C3	1.418 (3)	C14—C19	1.381 (3)
C3—C4	1.360 (3)	C14—C15	1.394 (3)
C3—H3	0.9500	C15—C16	1.378 (3)
C4—C5	1.430 (3)	C15—H15	0.9500
C4—H4	0.9500	C16—C17	1.383 (4)
C5—C6	1.417 (3)	C16—H16	0.9500
C5—C10	1.417 (3)	C17—C18	1.379 (4)
C6—C7	1.379 (4)	C17—H17	0.9500
C6—H6	0.9500	C18—C19	1.384 (3)
C7—C8	1.398 (4)	C18—H18	0.9500
C7—H7	0.9500	C19—H19	0.9500
			
Cg2···Cg3^i^	3.616 (4)		
			
O3—S1—O2	119.3 (1)	C11—C10—C5	114.7 (2)
O3—S1—C1	106.6 (1)	C11—C10—C9	124.3 (2)
O2—S1—C1	108.5 (1)	C5—C10—C9	121.0 (2)
O3—S1—C14	107.9 (1)	O1—C11—C2	110.6 (2)
O2—S1—C14	107.7 (1)	O1—C11—C10	124.5 (2)
C1—S1—C14	106.1 (1)	C2—C11—C10	124.9 (2)
C12—O1—C11	107.2 (2)	O1—C12—C1	110.2 (2)
C12—C1—C2	107.4 (2)	O1—C12—C13	115.3 (2)
C12—C1—S1	127.1 (2)	C1—C12—C13	134.5 (2)
C2—C1—S1	125.3 (2)	C12—C13—H13A	109.5
C11—C2—C3	119.1 (2)	C12—C13—H13B	109.5
C11—C2—C1	104.6 (2)	H13A—C13—H13B	109.5
C3—C2—C1	136.2 (2)	C12—C13—H13C	109.5
C4—C3—C2	118.2 (2)	H13A—C13—H13C	109.5
C4—C3—H3	120.9	H13B—C13—H13C	109.5
C2—C3—H3	120.9	C19—C14—C15	121.1 (2)
C3—C4—C5	122.4 (2)	C19—C14—S1	120.3 (2)
C3—C4—H4	118.8	C15—C14—S1	118.6 (2)
C5—C4—H4	118.8	C16—C15—C14	119.4 (2)
C6—C5—C10	117.6 (2)	C16—C15—H15	120.3
C6—C5—C4	121.8 (2)	C14—C15—H15	120.3
C10—C5—C4	120.6 (2)	C15—C16—C17	119.8 (2)
C7—C6—C5	120.8 (2)	C15—C16—H16	120.1
C7—C6—H6	119.6	C17—C16—H16	120.1
C5—C6—H6	119.6	C18—C17—C16	120.3 (2)
C6—C7—C8	120.5 (2)	C18—C17—H17	119.9
C6—C7—H7	119.7	C16—C17—H17	119.9
C8—C7—H7	119.7	C17—C18—C19	120.8 (2)
C9—C8—C7	121.1 (2)	C17—C18—H18	119.6
C9—C8—H8	119.4	C19—C18—H18	119.6
C7—C8—H8	119.4	C14—C19—C18	118.5 (2)
C8—C9—C10	119.0 (2)	C14—C19—H19	120.7
C8—C9—H9	120.5	C18—C19—H19	120.7
C10—C9—H9	120.5		
			
O3—S1—C1—C12	−142.38 (19)	C3—C2—C11—O1	−178.86 (17)
O2—S1—C1—C12	−12.7 (2)	C1—C2—C11—O1	0.0 (2)
C14—S1—C1—C12	102.8 (2)	C3—C2—C11—C10	0.2 (3)
O3—S1—C1—C2	32.34 (19)	C1—C2—C11—C10	179.12 (19)
O2—S1—C1—C2	162.06 (16)	C5—C10—C11—O1	179.07 (18)
C14—S1—C1—C2	−82.51 (18)	C9—C10—C11—O1	−1.2 (3)
C12—C1—C2—C11	−0.1 (2)	C5—C10—C11—C2	0.1 (3)
S1—C1—C2—C11	−175.74 (15)	C9—C10—C11—C2	179.8 (2)
C12—C1—C2—C3	178.5 (2)	C11—O1—C12—C1	−0.2 (2)
S1—C1—C2—C3	2.9 (3)	C11—O1—C12—C13	177.78 (17)
C11—C2—C3—C4	0.0 (3)	C2—C1—C12—O1	0.2 (2)
C1—C2—C3—C4	−178.5 (2)	S1—C1—C12—O1	175.70 (14)
C2—C3—C4—C5	−0.5 (3)	C2—C1—C12—C13	−177.2 (2)
C3—C4—C5—C6	−179.2 (2)	S1—C1—C12—C13	−1.7 (4)
C3—C4—C5—C10	0.9 (3)	O3—S1—C14—C19	−22.04 (19)
C10—C5—C6—C7	0.5 (3)	O2—S1—C14—C19	−152.05 (17)
C4—C5—C6—C7	−179.5 (2)	C1—S1—C14—C19	91.94 (18)
C5—C6—C7—C8	−0.2 (4)	O3—S1—C14—C15	156.92 (16)
C6—C7—C8—C9	−0.4 (4)	O2—S1—C14—C15	26.90 (19)
C7—C8—C9—C10	0.6 (3)	C1—S1—C14—C15	−89.10 (18)
C6—C5—C10—C11	179.43 (18)	C19—C14—C15—C16	1.4 (3)
C4—C5—C10—C11	−0.6 (3)	S1—C14—C15—C16	−177.59 (17)
C6—C5—C10—C9	−0.3 (3)	C14—C15—C16—C17	−1.0 (3)
C4—C5—C10—C9	179.69 (19)	C15—C16—C17—C18	−0.5 (4)
C8—C9—C10—C11	−179.9 (2)	C16—C17—C18—C19	1.6 (4)
C8—C9—C10—C5	−0.3 (3)	C15—C14—C19—C18	−0.2 (3)
C12—O1—C11—C2	0.1 (2)	S1—C14—C19—C18	178.69 (18)
C12—O1—C11—C10	−178.99 (19)	C17—C18—C19—C14	−1.2 (4)

Symmetry codes: (i) *x*−1/2, −*y*+1/2, *z*.

### Hydrogen-bond geometry (Å, °)

**Table:**

*D*—H···*A*	*D*—H	H···*A*	*D*···*A*	*D*—H···*A*
C13—H13A···Cg1^i^	0.98	2.64	3.483 (3)	144
C8—H8···O3^ii^	0.95	2.51	3.406 (3)	157
C16—H16···O3^iii^	0.95	2.51	3.430 (3)	164

Symmetry codes: (i) *x*−1/2, −*y*+1/2, *z*; (ii) −*x*+3/2, *y*+1/2, *z*−1/2; (iii) *x*−1, *y*, *z*.
